# Biomarker-guided duration of antibiotic treatment in children hospitalised with confirmed or suspected bacterial infection: statistical analysis plan for the BATCH trial and PRECISE sub-study

**DOI:** 10.1186/s13063-022-06956-9

**Published:** 2023-05-30

**Authors:** Simon M. Schoenbuchner, Chao Huang, Cherry-Ann Waldron, Emma Thomas-Jones, Kerenza Hood, Enitan D. Carrol, Philip Pallmann

**Affiliations:** 1 Centre for Trials Research, Cardiff University, Cardiff, UK; 2 Hull York Medical School, University of Hull, Hull, UK; 3 Institute of Infection, Veterinary and Ecological Sciences, University of Liverpool, Liverpool, UK

**Keywords:** Antimicrobial stewardship, Procalcitonin, Severe bacterial infection, Hospitalised children, Randomised controlled trial, Statistical analysis plan

## Abstract

**Introduction:**

The BATCH trial is a multi-centre randomised controlled trial to compare procalcitonin-guided management of severe bacterial infection in children with current management. PRECISE is a mechanistic sub-study embedded into the BATCH trial. This paper describes the statistical analysis plan for the BATCH trial and PRECISE sub-study.

**Methods:**

The BATCH trial will assess the effectiveness of an additional procalcitonin test in children (aged 72 h to 18 years) hospitalised with suspected or confirmed bacterial infection to guide antimicrobial prescribing decisions. Participants will be enrolled in the trial from randomisation until day 28 follow-up. The co-primary outcomes are duration of intravenous antibiotic use and a composite safety outcome. Target sample size is 1942 patients, based on detecting a 1-day reduction in intravenous antibiotic use (90% power, two-sided) and on a non-inferiority margin of 5% risk difference in the composite safety outcome (90% power, one-sided), while allowing for up to 10% loss to follow-up.

**Results:**

Baseline characteristics will be summarised overall, by trial arm, and by whether patients were recruited before or after the pause in recruitment due to the COVID-19 pandemic. In the primary analysis, duration of intravenous antibiotic use will be tested for superiority using Cox regression, and the composite safety outcome will be tested for non-inferiority using logistic regression. The intervention will be judged successful if it reduces the duration of intravenous antibiotic use without compromising safety. Secondary analyses will include sensitivity analyses, pre-specified subgroup analyses, and analysis of secondary outcomes. Two sub-studies, including PRECISE, involve additional pre-specified subgroup analyses. All analyses will be adjusted for the balancing factors used in the randomisation, namely centre and patient age.

**Conclusion:**

We describe the statistical analysis plan for the BATCH trial and PRECISE sub-study, including definitions of clinical outcomes, reporting guidelines, statistical principles, and analysis methods. The trial uses a design with co-primary superiority and non-inferiority endpoints. The analysis plan has been written prior to the completion of follow-up.

**Trial registration:**

BATCH: ISRCTN11369832, registered 20 September 2017, doi.org/10.1186/ISRCTN11369832.

PRECISE: ISRCTN14945050, registered 17 December 2020, doi.org/10.1186/ISRCTN14945050.

## Introduction

Most hospitals in the UK National Health Service (NHS) use a blood test called C-reactive protein (CRP) to monitor response to infection, but it is not specific for bacterial infection and shows a delayed response. Procalcitonin (PCT) is a blood test which is specific for bacterial infection and responds more quickly than CRP [1], but it is not routinely used in NHS. Previous studies, mostly in adults, show that using PCT to guide clinicians’ decision-making may reduce the amount of antibiotics used, reduce hospital stay, and is not associated with an increase in adverse effects such as hospital re-admission, incomplete treatment of infections, relapse, or death. A recent guideline from the National Institute for Health and Care Excellence (NICE) recommends further research on PCT testing to guide antibiotic use in children [2].

BATCH (biomarker-guided duration of antibiotic treatment in children hospitalised with confirmed or suspected bacterial infection) is a randomised controlled trial to compare PCT-guided management of severe bacterial infection in children with current management [3]. The primary research question is whether addition of PCT testing to current best practice based on the NICE antimicrobial stewardship guidelines [4] can safely allow a reduction in duration of antibiotic therapy in hospitalised children with suspected or confirmed bacterial infection compared to current best practice alone. The aim of the intervention is to reduce prescribing with no adverse effect on safety. Clinical management in the intervention arm is identical to current practice, but clinicians will have an additional PCT test with advice on how to interpret the result. Clinicians will use clinical judgement and may also use CRP to decide on duration of intravenous (IV) antibiotics.

PRECISE (MR-proADM [mid-regional pro-adrenomedullin] and ImmunoXpert evaluation of PCT-guided antibiotic duration in children with infection for stratification of effectiveness) is a mechanistic sub-study embedded into the BATCH trial. The aim of this sub-study is to determine if the effectiveness of the PCT-guided algorithm in reducing IV antibiotic duration varies depending on patients’ endothelial dysfunction (as measured by MR-proADM) and host immune response (as quantified by ImmunoXpert score).

This paper describes the statistical analysis plan (SAP) for the BATCH trial and PRECISE sub-study in advance of trial completion, including statistical principles, procedures for the analysis and presentation of primary and secondary trial outcomes, and pre-specified subgroup analyses.

### Co-primary outcomes

- Duration of IV antibiotics (h), derived from the starting and stopping times of IV antibiotic use
- Composite safety outcome with three components:
  1. Unscheduled admissions/readmissions to the paediatric intensive care unit (PICU) with/without infective diagnosis, or unplanned readmission to hospital, within 7 days of stopping IV antibiotics
  2. Restarting IV antibiotic therapy (for any reason) within 7 days of stopping IV antibiotics
  3. Mortality (death for any reason) in the 28 days following randomisation

### Secondary outcomes

- Each of the three components of the composite safety outcome
- Suspected adverse drug reactions (ADR), categorised using the Liverpool Causality Assessment Tool [5]
- Hospital-acquired infection (HAI) up to 28 days
- Total duration of antibiotic use (IV and oral), derived from the starting and stopping times of antibiotic use (hours)
- Time to switch from broad to narrow spectrum antibiotics (hours)
- Time to discharge from hospital (hours)

### Trial status

The BATCH trial recruited its first participant in June 2018. Recruitment was paused between March and May 2020 due to the coronavirus disease (COVID-19) pandemic. Recruitment of 1951 participants was completed on 12 October 2022, including over-recruitment to replace patients who were subsequently found to be ineligible. The SAP was drafted, and the manuscript submitted to the journal, before the final participant completed day 28 follow-up.

## Trial design

BATCH is a multi-centre, prospective, individually randomised, parallel, two-arm, controlled trial. The trial will assess the effectiveness of an additional PCT test in children (aged between 72 h and 18 years) hospitalised with suspected or confirmed bacterial infection to guide antimicrobial prescribing decisions.

Co-primary outcomes (antibiotic duration and a composite safety outcome) will be used to answer the primary research objectives of the study. Differences in antibiotic duration will be investigated for superiority of the intervention over standard care, while differences in the composite safety outcome will be investigated for non-inferiority [6].

### Intervention

In children randomised to the intervention arm, a blood sample will be sent to the hospital laboratory for a PCT test at baseline/randomisation and every 1–3 days while still on IV antibiotics to align with clinical workflow and routine laboratory testing where possible. PCT results feed into an algorithm [7] that provides both definitive and advisory guidelines, based on thresholds of PCT concentration and PCT change [8]. Clinicians can overrule the algorithm if they feel it is appropriate to do so. Children in the control arm will not have the PCT test performed. The trial protocol [3] includes further detail on the implementation of the intervention.

### Randomisation

Participants will be randomised in a 1:1 ratio to receive either current clinical management alone (control) or clinical management with the addition of PCT test guidance (intervention). Patients will be randomised by minimisation [9], with site and age group as minimisation factors and a random element to reduce predictability.

Participants will be randomised remotely using a secure 24-h web-based randomisation programme controlled centrally by the Centre for Trials Research at Cardiff University. Details of the age group cut-offs and random element are documented in a separate randomisation protocol and will be concealed from the treating teams.

### Sample size

The trial has two co-primary outcomes [6] (“Co-primary outcomes” section), and the overall sample size is determined by both. The focus for the intervention is on moving the step down from IV to oral therapy earlier, and therefore, the time until this step down is our primary outcome on antibiotic usage (overall usage across both oral and IV is a secondary outcome). The study is powered to detect if PCT-directed care is superior to standard care on time until switch from IV antibiotics. The size of potential shortening of time to detect an effect has been taken from a systematic review [10]. The safety co-primary outcome is a composite measure reflecting various outcomes which represent deterioration or lack of clinical response in the child, and it would therefore be expected to increase if IV antibiotics were being withdrawn inappropriately early.

A 1-day reduction [10] in IV antibiotic duration from an estimated median of 5 days [11] in the control arm implies a hazard ratio of 1.25 (assuming proportional hazards and an exponential survival distribution). At 5% two-sided significance level with 90% power, 844 participants with observed IV antibiotics duration are needed. In terms of the event rates of safety elements, we estimate an admission/re-admission rate of 8.8% [11]. In critically ill patients, up to 3% reinstating IV antibiotic therapy rate, and 4% mortality were reported [10, 12]. With some overlaps considered, we estimate an overall rate of about 15% for the composite safety outcome. A previous trial on PCT-guided antibiotic therapy in adults used a non-inferiority margin of 8% risk difference for mortality [13]. Given the lower expected rate of safety outcomes in this population, we have chosen a similar (relative) non-inferiority margin of 5% (absolute) risk difference for the composite safety outcome. This means that an increase of no more than 5% (from 15% to 20%) using PCT-guided therapy would be considered non-inferior. To test non-inferiority with a one-sided significance level of 0.05 and 90% power would therefore require 874 participants per arm. Overall, with 1748 effectively recruited participants, we would have 99% power to detect an antibiotic duration decrease corresponding to a hazard ratio of 1.25 and 90% power to test non-inferiority in safety separately. This means that the power of the combined analysis would be at least 89% (if the co-primary outcomes are independent) and at most 90% (if the co-primary outcomes are colinear) [14]. Allowing for up to 10% loss to follow-up, our target sample size is inflated to 1942.

### Data collection schedule

Participant data are collected at the following time points:

- At baseline (baseline characteristics and admission data)
- Daily post-randomisation until discharged home (antibiotic use, adverse events, and clinical data)
- Day 28 telephone follow-up (healthcare utilisation and quality of life questionnaire)

### Definition of non-adherence

There will be multiple measures of non-adherence, reflecting different stages of the clinical decision-making. Reasons for non-consideration of the PCT result or non-adherence to the algorithm can be broken down into three steps (Table 1). Steps 2 and 3 are only relevant when step 1 has been adhered to, i.e., when a PCT result is available. In cases where the PCT result was available and was considered, this will be considered adherence to the intervention policy specified in the trial protocol, regardless of the actual clinical decision [3].

**Table 1:** Types of non-adherence

Non-adherence step	Reasons or examples
1. PCT results not available	Blood samples not obtained, loss of IV access, blood sample insufficient for laboratory analysis, PCT machine issues, or results not available for ward rounds
2. PCT results not considered	If a PCT result was available, protocol requires that it be considered as part of clinical decision-making
3. PCT algorithm not adhered to	3a. If the PCT result was considered, protocol does not require clinicians to follow the PCT-guided algorithm. Clinical judgement may override the PCT-guided algorithm. Therefore, non-adherence to the PCT-guided algorithm is consistent with adherence to intervention policy 3b. If the PCT result was available but not considered, clinical judgement may or may not agree with the PCT-guided algorithm. Therefore, non-adherence to the PCT-guided algorithm will also be considered independently of adherence to the intervention policy

For those patients whose clinical reviews were adherent to the intervention policy (steps 1 and 2), we will investigate step 3a by comparing the actual clinical decision with the recommendation given by the PCT-guided algorithm. For patients whose procedures were adherent to step 1, we will separately investigate step 3b by comparing the actual clinical decision with the recommendation given by the PCT-guided algorithm, regardless of adherence to step 2.

In this clinical context, contamination of the control arm is very unlikely, i.e., we do not expect that any patient in the control arm will have a PCT test done.

### Analysis populations

All randomised participants will remain in their originally assigned groups, regardless of protocol deviations or non-adherence, and will be included in all analyses if outcome data are available.

In one of the planned secondary analyses (“Sensitivity analyses” section), we will estimate the complier average causal effect (CACE) [15] to account for departures from the randomised intervention. For the purposes of this sensitivity analysis, we will define different analysis populations depending on the level of adherence with the PCT-guided algorithm (as defined in the “Definition of non-adherence” section):

- Patients whose PCT result was available at the time of the clinical review
- Patients whose PCT result was available and was considered by the clinician (per protocol)
- Patients whose PCT result was available and was considered and where the actual clinical decision agreed/did not agree with the recommendation of the PCT-guided algorithm
  - ∘ Adherence to/overruling of recommendation to continue IV antibiotics
  - ∘ Adherence to/overruling of recommendation to stop IV antibiotics
- Patients whose PCT result was available and where the actual clinical decision agreed/did not agree with the recommendation of the PCT-guided algorithm, regardless of whether it was considered by the clinician

We will also consider adherence longitudinally (“Additional exploratory analyses” section). Most patients will have several PCT measurements taken at different points in time and availability/consideration/adherence may be different at different time points for the same child.

### Reporting

Final analysis of the primary and secondary outcomes will take place when all randomised participants have completed their day 28 telephone follow-up, all forms have been received, and the datasets have been locked. The trial report will follow the guidelines of Consolidated Standards of Reporting Trials (CONSORT) for reporting randomised controlled trials [16] and its extension to non-inferiority designs [17].

## Statistical principles

### Levels of confidence

To assess non-inferiority of the composite safety outcome, a one-sided 95% confidence interval will be calculated. Other outcomes will be assessed using two-sided 95% confidence intervals. Hypothesis tests will be conducted with type I error rate of 5%.

### Multiple testing

The trial has two arms, and no interim analyses are planned. The co-primary outcome will be assessed as an intersection–union test [18], meaning that we will consider the intervention successful if and only if both components are successful, i.e., if we conclude both non-inferiority of the composite safety outcome and reduction of IV antibiotic duration. No adjustment for multiplicity is necessary for the co-primary outcome; the intersection–union test requires rejection of both null hypotheses, so there is no inflation of the type I error rate [18]. We will correct for multiple hypothesis testing among the secondary and subgroup analyses by controlling the false discovery rate at 5% [19].

### Distributional assumptions

Modelling and distributional assumptions will be checked prior to reporting. Specifically, time-to-event models will be tested for the proportional hazard assumption, and logistic regression models will be assessed for overdispersion.

If distributional assumptions are not met, transformations will be attempted. If it is not possible to meet distributional assumptions via transformation, model choice may vary from what is described below. For example, a time interaction term may be added to Cox regression if the proportional hazard assumption is not met. Any changes will be fully documented.

### Statistical software

We will use Stata version 17 [20] for statistical analysis. R version 4 [21] will also be used for reporting and visualisation of results.

## Descriptive analyses

### Screening, eligibility, and recruitment

Identification of potential participants will be done by the clinical care team, or the clinical members of the research team involved in care of children on the ward, or the general paediatric or infectious diseases teams involved in care of children on the ward. A screening log will be kept at each site. Screening procedures are described in the trial protocol [3].

Children in whom antibiotics are likely to be continued for more than 48 h are potentially eligible for the trial. The clinician or designated research nurse will explain the trial to the child’s parent (used hereafter to refer to a person with legal responsibility for the child, who may be a carer designated as a legal guardian) and will ensure that they have had enough time to consider participation and answer any questions that they may have. Eligibility will be confirmed by a member the clinical care team, or delegated members of the research team, who may be medical or nursing practitioners. Detailed eligibility criteria are listed in the trial protocol [3]. Summary statistics on eligibility will be reported in the CONSORT diagram.

Procedures for informed consent and registration are described in the trial protocol [3]. Recruitment will be reported overall and by trial arm.

### Withdrawal and loss to follow-up

A participant may be withdrawn for the following reasons:

- Withdrawal of parental consent for the intervention, follow-up, data collection, and/or the entire trial
- Any alteration in the participant’s condition, which, in the opinion of the treating clinician, justifies the discontinuation of the intervention

Reasons for withdrawal will be reported in the CONSORT diagram. Withdrawals will be presented as frequencies and percentages, overall, and by trial arm. Participants will be identified as lost to follow-up if it is not possible to contact them for 6 weeks after randomisation. Loss to follow-up will also be reported in the CONSORT diagram.

### Baseline characteristics

The following data collected at baseline will be reported overall, by trial arm, and by whether patients were recruited before or after the pause in recruitment due to the COVID-19 pandemic:

- Age
- Sex
- Ethnicity
- Comorbidities: malignancy, pulmonary, gastrointestinal, neurological, cardiac, recent surgery, immunodeficiency, foreign body, allergy, endocrine, genetic, transplant, or other relevant history
- Route of admission: emergency department, inpatient ward, theatre, PICU, high-dependency unit, GP, or other hospital
- Duration of symptoms: number of hours before admission to hospital
- Prescribed antibiotic use during the past 14 days (including prophylactics)
- Initial diagnosis

Participant characteristics will be reported as frequencies and percentages, means and standard deviations, or medians and interquartile ranges, as appropriate. Baseline characteristics will be reported for all randomised participants, as well as for analysis populations as defined in the “Analysis populations” section. There will be no statistical comparison (e.g., using hypothesis tests) of baseline characteristics.

### Protocol deviations and non-adherence

Protocol deviations will be recorded, and frequencies will be presented overall and by trial arm. Where deviations occur, further details will be provided (e.g., reasons for non-eligibility). Potential protocol deviations will include:

- Participants who are randomised but do not meet eligibility criteria
- Participants randomised with incorrect date of birth
- Missed/late follow-up assessments
- Samples stored at incorrect temperature

Reasons for non-adherence will be broken down into the steps described above (Table 1). A patient may have multiple clinical reviews, so all three adherence steps will be recorded at each clinical review. Non-adherence will be presented, both overall and by trial arm, as frequencies and percentages, and visualised using a flow chart.

### Safety reporting

The trial population consists of hospitalised children. The primary composite safety outcome includes instances of the following events, to be recorded as part of routine data collection, and these will therefore *not* require expedited reporting as serious adverse events (SAEs):

- Death
- Life-threatening event
- Readmission to hospital or prolongation of hospitalisation

These adverse events will instead be recorded in participants’ notes and on the relevant case report forms (CRFs). The following events will be reported as SAEs within 24 h:

- Events resulting in persistent or significant disability or incapacity
- Congenital anomalies or birth defects

The following non-serious adverse events (AEs) will also be recorded as part of routine follow-up at 28 days:

- Non-serious AEs potentially attributable to PCT test and step-down approach
- Suspected drug reactions defined by the Liverpool Causality Assessment Tool

These events will be recorded in participants’ notes and on the relevant CRF. Other non-serious AEs will not be collected. (S)AEs will be categorised by seriousness and summarised by arm. The proportion of patients per arm experiencing each AE (if ≥ 2% of patients in either arm) and the risk ratio between arms will be presented graphically using a Cleveland dot plot [22].

## Primary analysis

The trial has two co-primary outcomes [6] (“Co-primary outcomes” section). We will compare the duration of IV antibiotic treatment between arms using Cox regression. We will use logistic regression to construct a one-sided confidence interval for the risk difference of the composite safety outcome via the delta method [23]. Non-inferiority will be concluded if the upper bound of the confidence interval is below + 5% on the risk difference scale. Trial arm and the minimisation factors will be included as covariates in both models, with centre as a random effect and age as a fixed effect. The intervention will be judged successful if and only if it is found to be both superior with respect to IV antibiotic duration and non-inferior with respect to the composite safety outcome (Table 2).

**Table 2:** Criteria for judging success of the intervention

	IV antibiotic duration differs between groups (H_1_)	IV antibiotic duration does not differ between groups (H_0_)
Safety composite not worse in PCT group (H_1_)	Intervention successful *if antibiotic duration is reduced in PCT group*	Intervention unsuccessful
Safety composite worse in PCT group (H_0_)	Intervention harmful	Intervention harmful

### Missing data

Missing data on the composite safety outcome is likely to be minimal, so complete case analysis will be used. Missingness (frequency and percentage) will be reported for each combination of the components of the composite safety outcome. For the primary analysis, the composite safety outcome will be considered missing if (1) data on unscheduled readmission are missing and (2) the patient is not known to have experienced at least one of the two other components. This assumes that it would be known if a patient had restarted IV antibiotics or died. The potential influence of this assumption will be investigated by comparing estimates from best-case and worst-case imputation (“Sensitivity analyses” section).

Missing data on IV antibiotic stopping time will lead to censoring at the patient’s last available clinical review. Patients with missing outcome data can therefore still be included in the Cox regression model, under the assumption that this censoring is non-informative. Truncation due to death would violate this assumption. The potential influence of this will be investigated in the survivor average causal effect (SACE) sensitivity analysis ( “Sensitivity analyses” section).

## Secondary analyses

Secondary and subgroup analyses of the co-primary outcomes are summarised in Table 3. Additional subgroup analyses are specified in two embedded sub-studies (“Sub-studies” section). The procedure for subgroup analyses is described in the “Subgroup analyses” section.

**Table 3:** Summary of analyses of the primary outcomes

Outcome	Analysis	Covariates
IV antibiotic duration	*Primary analysis:* Cox regression (superiority)	Trial arm and minimisation factors
	Kaplan Meier plot	Trial arm
	Log rank test	Trial arm
	*Sub-group analyses:* Cox regression (interaction test by model comparison)	Trial arm, minimisation factors, and organ system of infection (lower urinary, lower respiratory, intra-abdominal, bacteraemia, skin, soft tissue)
		Trial arm, minimisation factors, and whether patients were recruited before or after the pause in recruitment due to COVID-19
	Complier Average Causal Effect (CACE)	Trial arm, minimisation factors, and intervention adherence (“Analysis populations” section)
	Survivor Average Causal Effect (SACE)	Trial arm, minimisation factors, and mortality before stopping IV antibiotic use (“Sensitivity analyses” section)
Safety composite	*Primary analysis:* Logistic regression (non-inferiority)	Trial arm and minimisation factors
	*Sub-group analyses:* Logistic regression (interaction test by model comparison)	Trial arm, minimisation factors, and organ system of infection (lower urinary, lower respiratory, intra-abdominal, bacteraemia, skin, soft tissue)
		Trial arm, minimisation factors, and whether patients were recruited before or after the pause in recruitment due to COVID-19
	Complier Average Causal Effect (CACE)	Trial arm, minimisation factors, and intervention adherence (“Analysis populations” section)

### Subgroup analyses

We will perform subgroup analyses on the primary outcomes, only if the primary analysis of that outcome indicates that the intervention was successful (i.e., in the case of the composite safety outcome, if the intervention is found to be non-inferior). Subgroups will be based on pre-specified baseline characteristics (Table 3). The trial is not powered to reliably detect subgroup effects. Subgroup findings will be considered exploratory and will not affect the trial’s main conclusions.

In each sub-group analysis, we will investigate how the treatment effect varies between subgroups by adding the grouping variable as a covariate in the main analysis model, both with and without a treatment-arm interaction term. The models with and without the interaction will be compared using a likelihood-ratio test (LRT). We will report the LRT *χ*^2^ statistic and illustrate the direction of the subgroup effect using interaction plots.

Additional subgroup analyses are specified in two embedded sub-studies (“Sub-studies” section).

### Sensitivity analyses

The sample size calculation (“Sample size” section) assumed a 15% rate of the composite safety outcome in the control group. Because the non-inferiority margin is defined using a fixed risk difference, deviations from the assumed control group rate could cause a reduction in power (if > 15%) or an inflation of the tolerable relative risk in the treatment group (if < 15%). We will therefore repeat the primary analysis with the non-inferiority margin modified according to the power-stabilising arcsine transformation [24]. If the observed rate in the control group is less than 15%, we will also assess non-inferiority on the relative risk scale: the risk ratio will be calculated via the delta method [23], and non-inferiority will be concluded if the upper bound of the confidence interval is below ^4^/_3_. When the control group rate is less than 15%, this relative non-inferiority margin is more stringent than the absolute margin used in the primary analysis.

We will use CACE [15] to account for departures from the randomised intervention. CACE estimates the intervention effect for the subset of patients who would have received fully compliant treatment in either trial arm. In addition, primary analyses will be repeated on subpopulations based on different forms of non-adherence (“Analysis populations” section). If patients are recruited but subsequently found to have been ineligible, we will perform an additional sensitivity analysis excluding all ineligible patients.

We will use SACE [25] to account for the fact that IV antibiotic duration is undefined for patients who died before IV antibiotics were stopped. SACE estimates the intervention effect for the subset of patients who would have survived under either treatment.

To assess the impact of missing composite safety outcome data, we will compare the results of the primary analysis (complete cases, as defined in the “Missing data” section) with estimates from two simple imputation models. We will treat all missing components as negative (no event) in the “best-case” imputation, and as positive in the “worst-case” imputation, providing upper and lower bounds on the composite safety outcome.

### Analyses of secondary outcomes

Secondary outcomes will be analysed using logistic regression (binary outcomes) or Cox regression (duration outcomes). Trial arm and the minimisation factors will be included as covariates in each analysis, with centre as a random effect and age as a fixed effect.

### Additional exploratory analyses

The COVID-19 pandemic may lead to heterogeneous effects, for example via changes to the study population, changes in medical practice intervention, or as a direct result of COVID-19 infection [26]. We will investigate this possibility in one of the subgroup analyses described above. In addition, we will compare the effect of treatment between patients with and without a COVID-19 diagnosis. This exploratory analysis will be limited to patients recruited after the pause in recruitment, to ensure that patients with COVID-19 are compared only to concurrent controls.

We will investigate the overall quality of implementation and its clustering by site, with a focus on adherence as an indicator of clinician behaviour. We will classify clinicians by whether they adhere to the PCT-guided algorithm during least 90% of decisions, restricting the analysis to clinicians who have made a minimum number of decisions (this cut-off will be determined after inspection of the distribution of the number of decisions, whether adherent or not, taken per clinician). We will also explore how adherence changed over the course of the study and whether clinician choices were associated with the sex or ethnicity of the patient. Further exploratory implementation analysis may be added, based on the results of the qualitative process evaluation, which is described in the trial protocol [3].

Any analysis that is not specified in the SAP will be clearly identified as post hoc analysis in the final report or subsequent publications.

## Sub-studies

### PRECISE

The aim of the PRECISE sub-study is to identify whether the effectiveness of the PCT-guided algorithm in reducing IV antibiotic duration differs between patients with different endotypes, based on two biomarkers: MR-proADM, a marker of endothelial dysfunction [27, 28], and ImmunoXpert score, a marker of host immune response [29].

The PRECISE sub-study will take place at a subset of BATCH sites. Additional blood samples will be taken at randomisation, in both the intervention and the control arm. When extra blood samples are taken for routine care, then serial blood samples will also be taken for the PRECISE biomarkers, but no additional venepuncture or finger pricks will be performed.

Outcome definitions will be the same as in the host trial (BATCH). The primary outcome is duration of IV antibiotic treatment, and secondary outcomes are:

- Total duration of antibiotics (IV and oral)
- Time to switch from broad spectrum to narrow spectrum antibiotics
- Time to discharge from hospital
- Suspected ADR
- HAI up to 28 days

Sample size calculation for the PRECISE sub-study was based on a comparison between three subgroups, separately for each biomarker: In the sample size calculation for MR-proADM, we assumed median antibiotic durations of 2 versus 4 days (control vs. PCT) in the low MR-proADM subgroup, 7 versus 5 days (control vs. PCT) in the intermediate MR-proADM subgroup, and 10 versus 5 days (control vs. PCT) in the high MR-proADM subgroup. To detect an arm-by-subgroup interaction effect of this size with 90% power whilst controlling the type I error rate at 5%, we need 25 participants per biomarker subgroup in each trial arm [30]. To achieve this sample size, given the assumed proportions of participants falling into each MR-proADM subgroup, and inflating for 5% dropout, we need analysable baseline blood samples from 266 participants. In the sample size calculation for ImmunoXpert, we assumed median antibiotic durations of 1 versus 3 days (control vs. PCT) in the low ImmunoXpert subgroup, 7 versus 4 days (control vs. PCT) in the intermediate ImmunoXpert subgroup, and 10 versus 5 days (control vs. PCT) in the high ImmunoXpert subgroup. Based on similar calculations as for MR-proADM above, we need 13 participants per biomarker subgroup in each trial arm. To achieve this sample size, given the assumed proportions of participants falling into each ImmunoXpert subgroup, and inflating for 5% dropout, we would need analysable baseline blood samples from 138 participants. Consequently, 266 is the overall sample size target for this sub-study.

In the primary PRECISE analysis, we will fit a Cox proportional hazards model with duration of IV antibiotic treatment as dependent variable. We will test the interaction between biomarker score and treatment arm, separately for MR-proADM and for ImmunoXpert, using the procedure described for subgroup analyses in the “Subgroup analyses” section.

Analysis of the secondary outcomes will involve similar regression models, depending on the type of the outcome variable. In a further secondary analysis with IV antibiotic duration as the outcome, we will test the interaction between treatment arm and biomarker subgroups, categorised according to the following criteria: MR-proADM high (≥ 0.7 nmol/l) or low (< 0.7 nmol/l) [27], ImmunoXpert score high (> 0.65), intermediate, or low (< 0.35) [29, 31]. PRECISE secondary analyses will not be performed if analysis of the same outcome in the host trial fails to discover a significant treatment effect.

All PRECISE participants will remain in the trial arm assigned by randomisation in the host trial, regardless of protocol deviations or non-adherence, and will be included in all analyses if outcome and biomarker data are available. Missingness within the PRECISE sub-study will be reported for each outcome as described in the “Missing data” section. We will present a descriptive summary of serial biomarker measurements, including the distributions of the observation intervals and of the number of observations per patient.

### Comorbidities

A second sub-study embedded into the BATCH trial will examine the differential effect of the intervention in children with multiple long-term conditions. We will describe the frequency and percentage of the BATCH cohort with different types and numbers of comorbidities and present information on antibiotic use, clinical outcomes, and adherence to the PCT algorithm for each type and number of comorbidities.

The primary outcome is duration of IV antibiotic treatment, and secondary outcomes are the composite safety outcome and adherence to the PCT algorithm, as defined in the host trial (BATCH). All participants will remain in the trial arm assigned by randomisation in the main trial, regardless of protocol deviations or non-adherence, and will be included in the analysis if outcome and comorbidity data are available. Missing data on patient comorbidities is likely to be minimal.

For the primary analysis, we will fit a Cox proportional hazards model with duration of IV antibiotic treatment as dependent variable. Patients will be classified into three comorbidity subgroups (no comorbidity, single comorbidity, or multiple comorbidities). Secondary analyses will involve similar regression models, depending on the type of the outcome variable. The procedure for subgroup analyses is described in the “Subgroup analyses” section. Secondary analysis of the composite safety outcome in this sub-study will not be performed if the primary BATCH analysis finds that the intervention is inferior to standard care.

As an additional exploratory analysis, we will plot post-intervention PCT trajectories, stratified by subgroup. We will also explore the influence of respiratory comorbidities by further sub-dividing the comorbidity subgroups.

Trial documents

**Table:** 

	Version	Date
Protocol	5.1	5 May 2022
Statistical analysis plan	0.8	21 October 2022
Data management plan	3.0	5 November 2020
SOP/008/2 (Statistical analysis plan)	2.0	14 February 2022
SOP/008/4 (Statistical reporting)	1.3	28 October 2021

## Data Availability

Not applicable.

## Abbreviations

ADR: Adverse drug reaction
AE: Adverse event
BATCH: Biomarker-guided duration of antibiotic treatment in children hospitalised with confirmed or suspected bacterial infection
CACE: Complier average causal effect
CONSORT: Consolidated Standards of Reporting Trials
COVID-19: Coronavirus disease
CRF: Case report form
CRP: C-reactive protein
GP: General practitioner
HAI: Hospital-acquired infection
IV: Intravenous
LRT: Likelihood-ratio test
MR-proADM: Mid-regional pro-adrenomedullin
NHS: National Health Service
NICE: National Institute for Health and Care Excellence
NIHR: National Institute of Health Research
PCT: Procalcitonin
PICU: Paediatric intensive care unit
PRECISE: MR-proADM and ImmunoXpert evaluation of procalcitonin-guided antibiotic duration in children with infection for stratification of effectiveness
SACE: Survivor average causal effect
SAE: Serious adverse event
SAP: Statistical analysis plan
